# Effect of the Enrichment of Corn Oil With *alpha*- or *gamma*-Tocopherol on Its In Vitro Digestion Studied by ^1^H NMR and SPME-GC/MS; Formation of Hydroperoxy-, Hydroxy-, Keto-Dienes and Keto-*E*-epoxy-*E*-Monoenes in the More *alpha*-Tocopherol Enriched Samples

**DOI:** 10.3390/antiox9030246

**Published:** 2020-03-18

**Authors:** Jon Alberdi-Cedeño, María L. Ibargoitia, María D. Guillén

**Affiliations:** Food Technology, Faculty of Pharmacy, Lascaray Research Center, University of the Basque Country (UPV-EHU), Paseo de la Universidad nº 7, 01006 Vitoria-Gasteiz, Spain; jon.alberdi@ehu.es (J.A.-C.); marialuisa.ibargoitia@ehu.es (M.L.I.)

**Keywords:** corn oil, in vitro digestion, ^1^H NMR, SPME-GC/MS, *gamma*- and *alpha*-tocopherols, antioxidant, prooxidant, bioaccessibility

## Abstract

The aim of this study is the analysis of the in vitro digestion of corn oil, and of the effect of its enrichment with three levels of *gamma*- and *alpha*-tocopherol, by using, for the first time, ^1^H nuclear magnetic resonance (^1^H NMR) and a solid phase microextraction followed by gas chromatography/mass spectrometry (SPME-GC/MS). The attention is focused on the hydrolysis degree, the degradation of oil’s main components, the occurrence of oxidation reactions and main compounds formed, as well as on the bioaccessibility of oil’s main components, of compounds formed in the oxidation, and, of *gamma*- and *alpha*-tocopherol. The lipolysis levels reached are high and show a similar pattern in all cases. The oxidation of corn oil components during in vitro digestion is proven, as is the action of *gamma*-tocopherol as an antioxidant and *alpha*-tocopherol as a prooxidant. In the more *alpha*-tocopherol enriched samples, hydroperoxy-, hydroxy-, and keto-dienes, as well as keto-epoxy-monoenes and aldehydes, are generated. The bioaccessibility of the oil’s main components is high. The compounds formed in the oxidation process during in vitro digestion can also be considered bioaccessible. The bioaccessibility of *alpha*-tocopherol is smaller than that of *gamma*-tocopherol. The concentration of this latter compound remains unchanged during the in vitro digestion of the more *alpha*-tocopherol enriched oil samples.

## 1. Introduction

During gastrointestinal digestion, several reactions take place, which have important repercussions in food component bioaccessibility. The hydrolysis of proteins, carbohydrates and triglycerides is the main one, giving rise to smaller building blocks which are able to be absorbed. Some evidence suggests that other reactions may also be possible, such as the Maillard reaction, esterification and oxidation [1,2,3]. Of these, oxidation could be a cause for concern, because it is known that certain compounds resulting from lipid oxidation are toxic and have been held responsible for several degenerative diseases [4,5,6].

Nowadays, it seems to be well established that diets high in vegetable and fruit content are healthy, mainly due to these commodities being rich in compounds with antioxidant ability. For this reason, the enrichment of foods with compounds of natural origin with potential antioxidant ability is becoming common in the industry, for both technological and health reasons. In spite of this, the knowledge of the effect that this enrichment provokes in foods when they are submitted to different processes is scarce. Recently, it has been shown that some compounds, attributed to antioxidant ability, act as prooxidants in some foods, when submitted to certain oxidative conditions [7,8,9]. Taking into account all of the above mentioned, the study of the effect of the enrichment of lipid foods with compounds, with potential antioxidant ability on their behavior during digestion, is important for both industry and consumers. 

Vegetable oils, widely consumed around the world, are the quintessential lipid foods, and their enrichment with natural antioxidants may be considered of interest. The great number of natural compounds with potential antioxidant ability include tocopherols, which may be considered very suitable for enriching oils, due to their lipophilicity and because they are also minor components in vegetable oils. 

Nevertheless, so far, no study has been carried out on the effect of *gamma*-tocopherol enrichment on the digestion of lipid foods. With regard to the effect enrichment with *alpha*-tocopherol, some studies have been published; however, their results are not conclusive. Some of these studies have concluded that this latter enrichment does not cause changes in the digestion of lipid foods [10,11], or that the changes are not clear [12]. Others have found a decrease in the values of certain oxidation parameters, from which it follows that *alpha*-tocopherol acts as an antioxidant [2,12,13,14,15]. Finally, other ones have reported that *alpha*-tocopherol acts as a prooxidant [14,16]. These discrepancies between results may be due to several reasons, which may include: the kind of lipid food involved; the degree of enrichment in *alpha*-tocopherol; the digestion model employed; and the methodology used to evaluate the oxidation level of the digestate.

In this context, the aim of this study is to analyze the effect of enriching corn oil, with either *alpha*-tocopherol or with *gamma*-tocopherol, on its in vitro digestion. Corn oil is an edible oil consumed worldwide, and *alpha*- and *gamma*-tocopherol are two natural forms of vitamin E, to which antioxidant ability has been attributed. The study will simultaneously deal with various subjects such as: the lipolysis degree reached and the bioaccessibility of the compounds coming from oil main component hydrolysis; the occurrence of oxidation reactions during digestion and the nature of the main oxidation compounds formed, if any; the elucidation of the effect of enrichment with each tocopherol on the potential oil component oxidation during digestion; and, finally, *gamma*-tocopherol and *alpha*-tocopherol bioaccessibility, as well as the influence of the *alpha*-tocopherol added on the bioaccessibility of naturally present *gamma*-tocopherol in corn oil. Furthermore, in order to analyze the influence of tocopherol concentration on all the above mentioned issues, the study will be carried out with three different enrichment degrees of each tocopherol; it should be remembered that the current legislation allows one to add this kind of compounds to refined vegetable oils under the “*quantum satis*” principle. All of these matters will be studied using data obtained by^1^H nuclear magnetic resonance (^1^H NMR) spectra of the oil samples and of the lipid extracts of the digestates. Furthermore, solid phase microextraction (SPME), followed by gas chromatography/mass spectrometry (GC/MS), will be used, as a complementary technique, to ensure the occurrence and level of oxidation reached during digestion. This will be elucidated by the presence and abundance of oxidation markers in the headspace of the digestate. Although different digestion models can be used, a classical in vitro model, which has given satisfactory results for some time [17], will be used in this study; nevertheless our laboratory is in a process of change in this respect and we plan to use a consensus model in the future.

## 2. Materials and Methods 

### 2.1. Samples Subject of Study

The study was carried out with refined corn oil C, acquired in a local supermarket. Its composition in molar percentages of linolenic (Ln), linoleic (L), oleic (O) and saturated (S) acyl groups is, 0.6 ± 0.0%, 49.2 ± 0.5%, 34.1 ± 0.3% and 16.1 ± 0.1% respectively. This was determined from ^1^H NMR spectral data as in previous studies [18,19]. The tocopherols used were *alpha*-tocopherol (αT) (purity of 98.2%) purchased from Sigma-Aldrich (St. Louis, MO, USA), and *gamma*-tocopherol (γT) (purity of ≥ 90%) provided by Eisai Food & Chemical Co. Ltd. (Tokyo, Japan). Aliquots of the oil were enriched with *alpha*-tocopherol or *gamma*-tocopherol at 0.2%, 2% and 5% by weight in each case. The samples submitted to in vitro digestion were the original oil C, and all samples enriched in αT (C0.2αT, C2αT and C5αT) and in γT (C0.2γT, C2γT and C5γT). 

### 2.2. Digestion Experiments

Aliquots (0.5 g) of the above-mentioned samples were digested, following the semi-static in vitro gastrointestinal digestion model developed by Versantvoort, et al. (2005) [20]. This validated method was optimized, in order to improve the lipids digestion, attempting to reach lipolysis levels of a similar order to in vivo digestion [21]. It has three-stages which simulate digestive processes in mouth, stomach, and small intestine, by sequentially adding the corresponding digestive juices (saliva, gastric juice, duodenal juice and bile), of which the compositions is given in Table S1 (see Supplementary Material). The first stage begins by adding 6 mL of saliva to the sample. After 5 min of incubation, 12 mL of gastric juice is added and the mixture is rotated head-over-heels at 40 rpm for 2 h at 37 ± 2 °C. One hour after the start of the gastric stage, pH is set between 2 and 3 with HCl (37%), simulating the gradual acidification of the chyme occurring in vivo. After 2 h of the gastric stage, 2 mL of sodium bicarbonate solution (1 M), 12 mL of duodenal juice and 6 mL of bile juice are added. Subsequently, pH was set between 6 and 7, and the mixture is again rotated at 40 rpm and incubated at 37 ± 2 °C for 4 h. All the reagents and enzymes for the preparation of digestive juices were acquired from Sigma-Aldrich (St. Louis, MO, USA): α-amylase from *Aspergillus oryzae* (10065, ~30 U/mg); pepsin from porcine gastric mucosa (P7125, ≥400 U/mg protein); amano lipase A from *Aspergillus niger* (534781, ≥120,000 U/g); pancreatin from porcine pancreas (P1750); lipase type II crude from porcine pancreas (L3126, 100–500 U/mg protein (using olive oil, 30 min incubation)) and bovine bile extract (B3883). The digested samples were named after the original samples, but preceded by D (DC, DC0.2αT, DC2αT, DC5αT, DC0.2γT, DC2γT and DC5γT). Three digestion experiments, each including duplicate samples, were performed. Blank samples corresponding to the mixture of juices submitted to digestive conditions were also taken for further analysis.

### 2.3. Digestate Lipid Extraction

Lipids of the digestates were extracted using dichloromethane as solvent (CH_2_Cl_2_, HPLC grade, Sigma-Aldrich, St. Louis, MO, USA), following a methodology that also allows fatty acid extraction as in previous studies [22]. This methodology involves a three-stage liquid-liquid extraction process, with 20 mL of CH_2_Cl_2_ each. Afterwards, to ensure the complete protonation of fatty acids and/or the dissociation of the potential salts formed, the remaining water phase was acidified to pH 2 with HCl (37%) and a second extraction was carried out in three steps. All the CH_2_Cl_2_ extracts of each sample were mixed and any solvent was eliminated by means of a rotary evaporator under reduced pressure at room temperature, in order to avoid lipid oxidation. The extraction yield was, in all cases, near 85%. These extracts contain triglycerides, diglycerides and monoglycerides, as well as fatty acids and tocopherols and other minor lipophilic compounds, either present in the original oil samples or formed from oil components in the digestion process.

### 2.4. Study by ^1^H NMR of Oil Samples and Lipid Extracts of Digestates

#### 2.4.1. Operating Conditions

The ^1^H NMR spectra of the original oil C, and of the oil samples enriched with each one of the tocopherols at the different concentrations (C0.2αT, C2αT, C5αT; C0.2γT, C2γT and C5γT), and of the lipids extracted from their digestates (DC, DC0.2αT, DC2αT, DC5αT, DC0.2γT, DC2γT and DC5γT), were acquired in duplicate using a Bruker Avance 400 spectrometer operating at 400 MHz. For this purpose, the above-mentioned samples (approximately 0.16 g) were dissolved in 400 µL of deuterated chloroform, which contained tetramethylsilane (TMS), as an internal reference (Cortec, Paris, France). The acquisition conditions were the same as those used in previous studies [23]. It must be noted that the relaxation delay and acquisition time allow the complete relaxation of the protons, the signal areas thus being proportional to the number of protons that generate them, making it possible to use them for quantitative purposes.

#### 2.4.2. Identification of the Components

**Figure 1:**
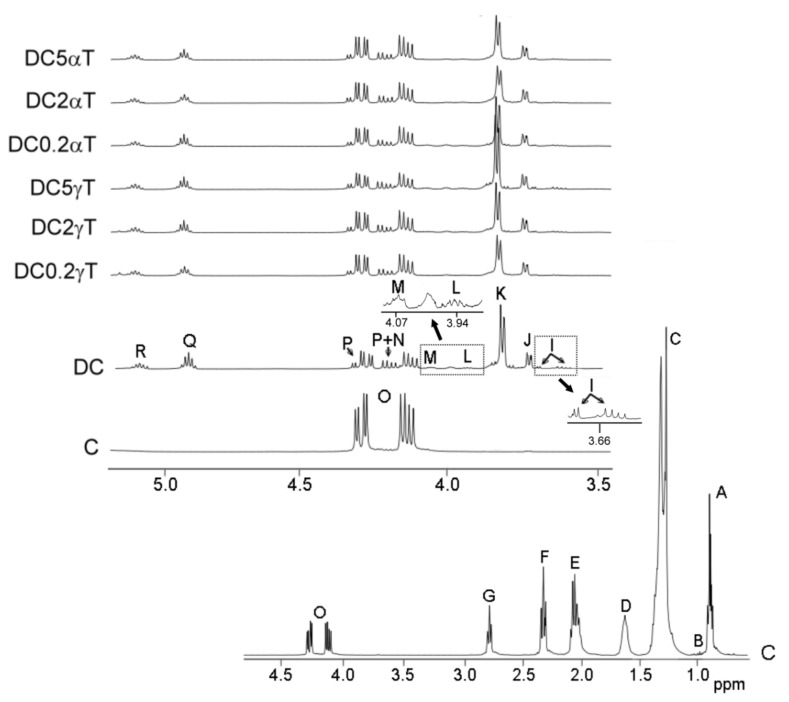
Region comprised between 0.0 and 4.9 ppm, of corn oil C ^1^H NMR spectrum, and region comprised between 3.5 ppm and 5.10 ppm, conveniently enlarged, of the ^1^H NMR spectra of the oil C and of the lipids extracted from the several digestates (DC, DC0.2γT, DC2γT, DC5γT and DC0.2αT, DC2αT, DC5αT), in which signals of protons of their main components appear. The signal letters agree with those of Tables S2 and S3.

**Figure 2:**
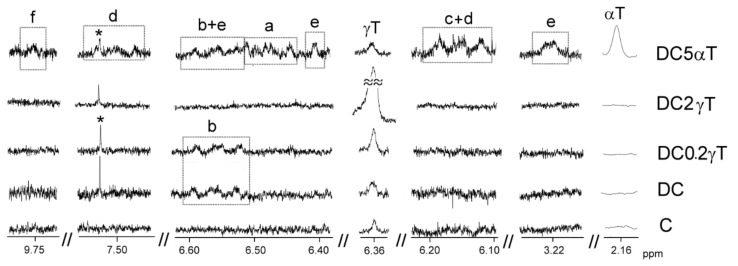
Some regions of the ^1^H NMR spectra of oil C and of the lipids extracted from the digestates of some enriched in tocopherol samples, conveniently enlarged, where signals of protons belonging to oxidation compounds and to *gamma*- and to *alpha*-tocopherol appear. The signal letters agree with those of Tables S4 and S5. The singlet marked with * is a satellite peak of chloroform.

The identification of the components present in the original oil, in the oil samples enriched with tocopherol and in the lipid extracts of their digestates, was carried out on the basis of the assignments of the ^1^H NMR signals, present in Figure 1; Figure 2, to the different kinds of hydrogen atoms, and to the different compounds. 

These signals, their chemical shifts and assignments are given in Tables S2–S5 (Supplementary Material). Their assignments were made, taking into account previous studies as indicated in each table, or on the basis of the signals of standard compounds acquired for this study. Among the latter are: *trans*-12,13-epoxy-9-keto-10(*E*)-octadecenoic acid, linolein hydroperoxides, linolein hydroxides, 9-oxo-10*E*,12*Z*-octadecadienoic acid and 13-oxo-9*Z*,11*E*-octadecadienoic acid purchased from Cayman Chemical (Ann Arbor, MI, USA), 9(S)-Hydroxy-10(*E*),12(*E*)-octadecadienoic acid (Dimorphecolic acid), acquired from Larodan (Malmö, Sweeden). 

Table S2 shows ^1^H NMR signals of specific protons of the different glyceride structures, such as triglycerides, diglycerides and monoglycerides. Table S3 shows the ^1^H NMR signals of protons of linolenic, linoleic, oleic and saturated acyl groups and fatty acids, and the signals of methylenic protons supported on carbons atoms in *alpha* position, in relation to carbonyl-carboxyl groups. Table S4 shows ^1^H NMR signals of protons of oxidation compounds coming from the main oil components degradation, which occurred during digestion. Finally, Table S5 gives ^1^H NMR signals of protons of *alpha*- and *gamma*-tocopherol. The areas of some of these spectral signals were used to quantify the concentration of the different kinds of the above-mentioned structures in the corresponding samples, as it will be explained below. 

#### 2.4.3. Quantifications Made from ^1^H NMR Spectral Data

This technique allows the estimation of the concentrations, expressed in different ways, of all the identified compounds mentioned above. This is possible because, as has been explained above, the area of the ^1^H NMR signals is proportional to the number of protons that generate the signal. The quantification of the different kinds of compounds or structures is explained below.

(A) Estimation of the Molar Percentage of the Different Kinds of Glycerides in the Digestates

The estimation of the molar percentage of each kind of glyceride structures can be carried out, by using the intensity of some signals indicated in Tables S2 and S3, which can also be observed in Figure 1. Although glycerol is formed during digestion, due to its polar nature, it is not present in the lipid extract of the digestates. However, its concentration can be estimated indirectly. This is possible because the concentration of total fatty acids plus acyl groups, of only acyl groups, and of fatty acids released in the formation of diglycerides and monoglycerides can be determined from ^1^H NMR data. Thus, the estimation of the molar percentage of triglycerides (TG), 1,2-diglycerides (1,2-DG), 1,3-diglycerides (1,3-DG), 2-monoglycerides (2-MG), 1-monoglycerides (1-MG) and glycerol (Gol), in relation to the total glyceryl structures present in the digestate, was carried out using equations [eq. S1–eq. S10] given in Supplementary Material. They are based exclusively on the area of ^1^H NMR spectral signals [24].

(B) Estimation of the Percentage of Fatty Acids Plus Acyl Groups that have Linoleic Structure in Relation to the Total of all Types of Fatty Acids and Acyl Groups in Digestates

In refined oils, the concentration of fatty acids is very small and unappreciable in comparison with the concentration of acyl groups. However, as is known, hydrolysis during oil digestion provokes the transformation of a certain number of acyl groups into fatty acids. The fatty acids formed maintain the same number of carbon atoms and unsaturation pattern as the starting acyl groups. Acyl groups and fatty acids having the same structure provide NMR spectra signals with a high degree of overlapping, which allows their joint quantification. In this study, the molar percentage of the linoleic acyl groups plus linoleic fatty acids in the digestates was estimated, in relation to the total number of moles of all kinds of fatty acids plus acyl groups. This estimation was made using the equation [eq. S11], given in Supplementary Material, in which the areas of some signals that are shown in Figure 1 and in Table S3 are involved. This equation is the same as employed in previous studies [18,19], but using the signal of methylenic protons supported on carbon atoms in *alpha* position in relation to carbonyl-carboxyl groups, instead of the signal of triglyceride protons used in edible oil studies.

(C) Estimation of the Concentration of Specific Compounds (SC) in Oil Samples and in the Digestates

The concentration of oxidation compounds, and of other ones, such as *gamma*- and *alpha*-tocopherol, either in oils or in digestates, can be estimated by using the general equation [eq. S12] given in Supplementary Material and the intensity of one of their non-overlapped ^1^H NMR spectral signal, which are shown in Figure 1 and Figure 2, in Figure S1 and in Tables S3, S4 and S5. This equation allows one to estimate the concentration of any compound in oils or in digestates in relation to the concentration of fatty acids plus acyl groups, which are considered the internal reference.

### 2.5. Study by SPME-GC/MS of the Headspace of Digestates and of the Mixture of the Digestive Juices Submitted to Digestion Conditions with the Corn Oil 

The extraction of the volatile components constituting the headspace of several samples (0.5 g in a 10 mL screw-cap vial) was carried out automatically using a CombiPAL autosampler (Agilent Technologies, Santa Clara, CA, USA). The samples studied were several digestates (DC, DC0.2αT, DC2αT, DC5αT, DC0.2γT, DC2γT and DC5γT) and the mixture CDJ of digestive juices DJ, after submission to digestion conditions, and corn oil C. The comparison of the headspaces of the several samples enables one to deduce differences provoked in them by in vitro digestion.

The fiber used for the headspace components extraction was coated with Divinylbenzene/Carboxen/Polydimethylsiloxane (DVB/CAR/PDMS, 50/30 μm film thickness, 1 cm long; acquired from Supelco (Sigma-Aldrich, St. Louis, MO, USA)). It was inserted into the headspace of the sample and maintained for 55 min at 50 °C, after a pre-equilibration time of 5 min. The fiber containing the components extracted was desorbed for 10 min in the injection port (splitless mode with 5 min purge time) of a 7890A gas chromatograph, equipped with a 5975C inert MSD with a Triple Axis Detector (Agilent Technologies, Palo Alto, CA, USA) and a computer operating with the ChemStation program. A fused silica capillary column was used (60 m length, 0.25 mm inside diameter, 0.25 μm film thickness; from Agilent Technologies Inc., Palo Alto, CA, USA), coated with a nonpolar stationary phase (HP-5MS, 5% phenyl methyl siloxane). The operation conditions were the following: the injector and interface temperatures were held at 250 °C and 305 °C respectively, and helium at a constant pressure of 117 kPa (16.9 psi) was used as the carrier gas. The oven temperature was initially held at 50 °C for 5 min, increased from 50 to 300 °C at a rate of 4 °C/min, and then held at 300 °C for 30 min. Mass spectra were recorded at an ionization energy of 70 eV, with data acquisition in Scan mode. The temperatures of the ion source and the quadrupole mass analyzer were kept at 230 and 150 °C, respectively. A reference sample of known composition was periodically analyzed, in order to verify the sensitivity of the SPME-GC/MS experiments, as in previous studies [25].

Identification of the headspace components was performed using several commercial standard compounds, acquired from Sigma-Aldrich (St. Louis, MO, USA). When standard compounds were not available, the identification was made by matching the spectra obtained, higher than 85%, with those of commercial libraries (Wiley W9N08, Mass Spectral Database of the National Institute of Standards and Technology) or with those spectra provided by the scientific literature, as in previous studies [25].

Semi-quantification of the compounds was based on the area counts of the base peak (Bp) of the mass spectrum of each compound, divided by 10^6^. When the Bp of a compound overlapped with some ion peak of the mass spectrum of another compound, an alternative ion peak was selected for the semi-quantification of the former [25]. Although the chromatographic response factor of each compound is different, the area counts thus determined are useful for the comparison of the abundance of each compound in the different samples. The detection limit was established at an abundance of 50,000 area counts. Data given in the following tables are average values of duplicate experiments.

### 2.6. Statistical Analysis 

The significance of the differences in the several kinds of data among samples was determined by a one-way variance analysis (ANOVA) followed by Tukey *b* test at *p* < 0.05, using SPSS Statistics 24 software (IBM, NY, USA).

## 3. Results

### 3.1. Extent and Pattern of Lipolysis Produced by the In Vitro Digestion in the Several Samples

As is known, the main components of the edible oils are triglycerides, and when they are submitted to digestion, hydrolysis of their ester bonds occurs, yielding diglycerides and monoglycerides, as well as fatty acids and glycerol. For this reason, in the ^1^H NMR spectral region shown in Figure 1 of corn oil C, almost the only signal observable, due to glycerides, is the signal O of triglyceride protons. Nevertheless, in the same spectral region of the lipid extract of the corn oil digestates (DC, DC0.2γT, DC2γT DC5γT, DC0.2αT, DC2αT and DC5αT), which is also shown in Figure 1, signals J, P and R of 1,2-diglicerydes, signals K and Q of 2-monoglycerides, signal M of 1,3-diglycerides, and signals I, L and N of 1-monoglycerides, are clearly observable. In addition, signal O of triglycerides also appears, but in much lower intensity than in the oil C spectrum. This indicates that triglyceride hydrolysis has taken place during this digestion. The extent of the hydrolysis can be inferred from the molar percentages of each kind of glyceride structure in relation to the total. These molar percentages were estimated, in all these samples, by means of the equations [eq. S1–eq. S10], by using the intensity of some signals given in Tables S2 and S3 and shown in Figure 1, as described in the Supplementary Material. The results obtained are given in Table 1.

(1) Lipolysis extent in corn oil digestate. It may be observed in Table 1 that the lipolysis extent provoked by the in vitro digestion in corn oil C is fairly high. About 78% of triglycerides have been hydrolyzed partially or totally. Monoglycerides and glycerol are the main glycerides formed (the yield of each one of these two kinds of compounds is approximately 31%); however, only about 15% of the triglycerides have been transformed into diglycerides. In general terms, this hydrolysis pattern is similar to that previously found in other edible oils submitted to the same digestion model [26].

(2) Lipolysis extent in the enriched in tocopherol corn oil digestates. As the data in Table 1 show, the triglyceride hydrolysis pattern of these samples is very similar to that found in the digestate of corn oil: monoglycerides and glycerol are the main hydrolytic products, followed by diglycerides, whose concentration is approximately half that of the other two hydrolytic products. Statistical treatment finds no significant differences between the lipolysis pattern and the extent occurred during in vitro digestion of the original oil and of the tocopherol enriched samples. Nevertheless, the results suggest that enrichment with tocopherols could have had some influence on the hydrolysis extent. Data in Table 1 could suggest that a slightly higher degree of hydrolysis took place in samples enriched with *gamma*-tocopherol, than it did in the unenriched oil. However no conclusive results were found in the samples enriched with *alpha*-tocopherol. This potential small effect in the tocopherol-enriched samples could be attributed to, among other reasons, to the different interactions that these compounds are able to establish with the components of the complex mixture involved in the digestion. It must be remembered that these two tocopherols have structural differences that translate into differences in polarity and lipophilicity etcetera, and as a consequence in differences in their behavior [27].

### 3.2. Bioaccessibility of Oil Main Components

The bioaccessibility of a compound or a group of compounds has been defined as the quantity or fraction of this compound or of the group of compounds, which is released from the food matrix into the gastrointestinal tract and becomes available for absorption [28]. As mentioned before, several reactions take place during digestion, and consequently, the food components undergo modifications in such a way that the amount of food components absorbable after digestion is different from the amount present in the original food. The methodology used in this study allows one to determine the bioaccessibility of the oil main components.

The only compounds released during digestion as a result of triglyceride hydrolysis which can be absorbed by enterocytes of the intestinal wall are fatty acids and monoglycerides. For this reason, the bioaccessibility of oil main components (B_OMC_), which is totally dependent on the extent and pattern of the hydrolysis occurring during digestion, is defined by the ratio between the concentration of compounds really absorbable present in the digestate, which are fatty acids plus monoglycerides ([FA] + [MG])_D_ and the concentration of all potentially absorbable compounds before digestion, which coincides with that of fatty acids plus all acyl groups ([FA] + [AG])_D_, as indicated in the following equation: B_OMC_ = ([FA] + [MG])_D_/([FA] + [AG])_D_. All these concentration data can be estimated from the intensities of certain ^1^H NMR signals of the corresponding samples, by using the equations given in the Supplementary Material. The data obtained are given in Table 1.

These data indicate that the bioaccessibility of these compounds is around 0.70 mol([FA] + [MG])_D_/mol([FA] + [AG])_D_ not only in the corn oil digestate, but also in the digestates of the oil samples enriched in tocopherols. That is to say, from every hundred original acyl groups supported in the oil triglycerides, nearly seventy are transformed, either into fatty acids or into monoglycerides, during digestion, and for this reason are available for absorption. As in the case of the molar percentages of the different kinds of glyceryl structures, the differences found between the bioaccessibility of oil main components in the original oil and in that enriched with tocopherol are not statistically significant. However, the data may suggest that enrichment with *gamma*-tocopherol could slightly favor the bioaccessibility of corn oil triglycerides, whereas enrichment with *alpha*-tocopherol could slightly reduce this bioaccessibility.

### 3.3. Study of the Occurrence of Oxidation Reactions during In Vitro Digestion of Corn Oil and of Corn Oil Samples Enriched in Tocopherols

As mentioned in the introduction, the occurrence of oxidation reactions during in vitro digestion has been described previously [1,2]. In a lipid system, such as corn oil, it is well accepted that oxidation first provokes the progressive degradation of its unsaturated acyl groups [29]. This degradation yields the so-called primary oxidation compounds, which evolve to give rise to the formation of secondary oxidation compounds of very varied nature and size. The study of the oxidation process of edible oils can be tackled by means of very different methodologies and techniques. Nevertheless, there are two that have great interest, not only by their efficiency, versatility, simplicity and rapidity, but also because they are very environmentally friendly and do not provoke any chemical modification of the sample, namely ^1^H NMR spectroscopy and SPME-GC/MS. The first allows detection of the occurrence of oxidation reactions, not only because it is able to quantify the concentration of the different kinds of acyl groups and fatty acids in oil or in lipid extracts, but also because it is able to detect, identify, and quantify the compounds formed in oxidation processes. SPME-GC/MS is much more sensitive than ^1^H NMR and allows one to identify and semi-quantify only volatile components, among which there may be oxidation markers. For all these reasons, both techniques were used to study the potential occurrence of oxidation reactions during in vitro digestion.

#### 3.3.1. Information Provided by ^1^H NMR Spectroscopy about the Occurrence of Oxidation Reactions during In Vitro Digestion 

As mentioned, two main procedures take place during oxidation, namely the degradation of unsaturated acyl groups and unsaturated fatty acids and the formation of compounds derived from them. This technique is able to evaluate both.

- Evaluation of the Concentration of Acyl Groups and Fatty Acids Having Linoleic Structure in the Corn Oil, in Its Digestate and in Those of Corn Oil Samples Enriched in Tocopherols

Linoleic is the main unsaturated acyl group in corn oil. After hydrolysis provoked by in vitro digestion, linoleic structures of acyl groups and of fatty acids are also the main unsaturated lipid structures in the digestates. For this reason, analysis of the concentration of this unsaturated structure, both in corn oil and in the lipid extracts of several digestates, will provide information concerning the occurrence of its degradation during in vitro digestion.

As before mentioned, linoleic structures of fatty acids and acyl groups give ^1^H NMR signals, very or totally overlapped, for which reason their concentration can be determined jointly. This can be carried out by using equation [eq. S11], given in Supplementary Material. The data obtained, given in molar percentage in relation to the total fatty acids plus acyl groups, are shown in Table 2.

From these data, it is evident that a significant and clear reduction of the molar percentage of linoleic structures occurs during the corn oil in vitro digestion, which evidences their degradation. However, in the samples enriched with gam*ma*-tocopherol, this reduction is significantly smaller than in the non-enriched oil. It can be inferred from these results that this compound has a protective effect against the degradation of linoleic structures. Finally, the loss of linoleic structures in the digestion of the samples enriched with *alpha*-tocopherol is slightly higher than that found in the non-enriched oil, although the differences are not statistically significant. Taking into account that the main cause of degradation of these unsaturated structures is oxidation, it could be thought that the in vitro digestion of corn oil provokes its oxidation and that while *gamma*-tocopherol reduces this degradative process, *alpha*-tocopherol could have the opposite effect. If lipid oxidation reactions have taken place during in vitro digestion, primary or secondary oxidation compounds, or both, should have been generated and they could be detected and quantified by ^1^H NMR spectroscopy.

- Study of the Formation of Oxidation Compounds during in vitro Digestion of Corn Oil and of Corn Oil Samples Enriched in Tocopherols

The first oxidation compounds formed in any oil oxidation process are hydroperoxydes, and when they come from linoleic groups, as is the case here, these hydroperoxides are supported on chains which also have conjugated dienic systems. These give well known signals in the ^1^H NMR spectra [29]. The degradation of these intermediate compounds generates secondary oxidation compounds of very varied nature. Among these latter can be cited hydroxy-dienes [30] and keto-dienes [7], with known ^1^H NMR spectral signals. In addition, other derived compounds having much more complex structures can also be formed, such as keto-epoxy-monoenes [8] and other ones formed in the rupture of secondary oxidation compounds [31] such as aldehydes.^1^H NMR spectral signals of all these compounds are given in Table S4. Their concentration can be estimated from the intensity of some of their signals in the spectrum and using equation [eq. S12] as is indicated in point 2.4.3, C.

**Figure 3:**
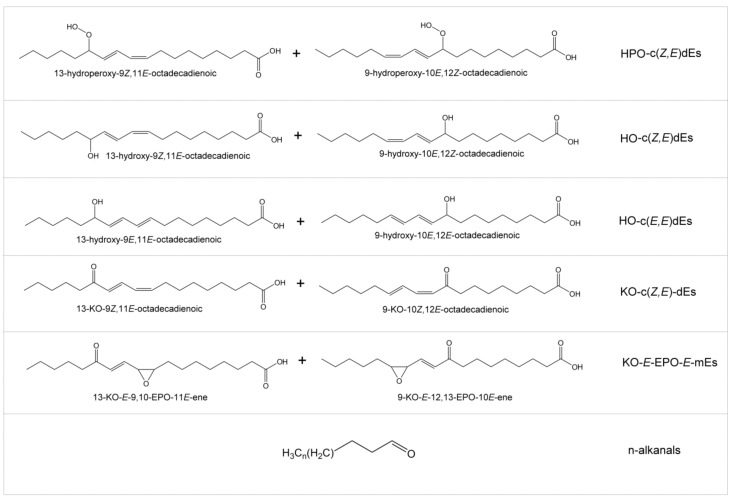
Chemical structures of some potential oxidation compounds present in the digestates of the corn oil samples enriched in *alpha*-tocopherol.

(1) Oxidation compounds in corn oil digestate. As Figure 2 shows, the^1^H NMR spectrum of the corn oil (C) has no signals of protons of oxidation compounds. This indicates that this oil has not been oxidized. However, the spectrum of the lipid extract of its digestate (DC) contains signals of protons of hydroperoxy-conjugated-(*Z,E*)-dienes (HPO-c(*Z,E*)dEs) (see Table S4 and Figure 2). The concentration of these compounds in DC is given in Table 2. This is clear evidence that in vitro digestion provokes the oxidation of corn oil generating this specific kind of primary oxidation compound.

(2) Oxidation compounds in the digestates of corn oil enriched in *gamma*-tocopherol. The ^1^H NMR spectrum of the lipid extract of the digestate of corn oil enriched with *gamma*-tocopherol at the lowest concentration essayed (DC0.2γT) also contains signals of hydroperoxy-conjugated-(Z,*E*)-dienes (HPO-c(*Z,E*)dEs) (Figure 2) but, as Table 2 shows, in smaller concentrations than in DC. It is noteworthy that, in the ^1^H NMR spectra of the extracts of the digestates DC2γT and DC5γT, no signals of hydroperoxy-conjugated-(*Z,E*)-dienes, nor those of any other oxidation compound, are present (Table 2 and Figure 2). This confirms that *gamma*-tocopherol acts as an antioxidant during this in vitro digestion, reducing the oxidation of corn oil (at an enrichment of 0.2%), or avoiding it totally (at enrichments of 2% and 5%).

(3)Oxidation compounds in the digestates of corn oil enriched in *alpha*-tocopherol. The effect provoked by the enrichment with *alpha*-tocopherol is the opposite of that provoked by the enrichment with *gamma*-tocopherol. The lipid extract of the digestates of all samples enriched with the first compound have a higher oxidation degree than DC. The ^1^H NMR spectrum of the extract of DC0.2αT only shows signals of hydroperoxy-conjugated-(*Z,E*)-dienes(HPO-c(*Z,E*)dEs), but as Table 2 shows, at a higher concentration than in the extract of DC. At higher concentrations of enrichment with *alpha*-tocopherol, the effect is even clearer. As can be observed in Figure 2, not only are there signals of hydroperoxy-conjugated-(*Z,E*)-dienes (HPO-c(*Z,E*)dEs) in the spectra of extracts of DC2αT and DC5αT, but also those of secondary and further oxidation compounds. These latter include signals of hydroxy-conjugated-(*Z,E*)-dienes, (HO-c(*Z,E*)dEs), such as those of hydroxy-conjugated-(*E,E*)-dienes (HO-c(*E,E*)dEs), such as of keto-conjugated-(*Z,E*)-dienes (KO-c(*Z,E*)dEs), as well as of keto-(*E*)-epoxy-(*E*)-monoenes (KO-*E*-EPO-*E*-mEs) and of saturated aldehydes. The concentration of primary oxidation compounds, in both samples DC2αT and DC5αT, is only slightly higher than in DC, but these samples contain other secondary and further oxidation compounds in even higher concentrations than those of the primary oxidation compounds, as shown in Table 2. Figure 3 shows some of the oxidation compounds with the above-mentioned structures.

These results may lead to several observations:

(a) In spite of *alpha*-tocopherol and *gamma*-tocopherol being known as two forms of Vitamin E, their behaviour is totally different during in vitro digestion. The first acts as a prooxidant at the three concentrations essayed, in agreement with some previous studies [14,16], whereas the second acts as an antioxidant. This difference in behaviour has been also observed in studies of edible oils enriched in these compounds submitted to conditions of accelerated storage. In these studies, *alpha* tocopherol exhibits a prooxidant effect, which increases in line with its concentration, whereas *gamma*-tocopherol only accelerates oxidation at very early oxidation stages. However, the global effect in the total oxidation process could be considered antioxidant [7,8,9].

(b) Elucidation of the behaviour of a compound as either antioxidant or prooxidant during digestion cannot be made exclusively on the basis of the estimation of a parameter such as peroxide value or other equivalents. In this study, the concentration of hydroperoxides is fairly similar in DC and DC5αT, as Table 2 shows, and from these data it could be concluded that *alpha*-tocopherol has no great influence on the oxidation occurred during digestion, against the evidence.

(c) In in vitro digestion, which takes place at 37 °C, oil oxidation advances very quickly in comparison with experiments on corn oil, or on other oils of similar composition, submitted to accelerated storage conditions at 70 °C. In the in vitro digestion of this corn oil, primary and secondary or further oxidation compounds are present in the digestates after 6 h and in concentrations of a similar order. However, in experiments with corn oil, or with other oils which are rich in linoleic groups, submitted to accelerated storage conditions, hydroperoxides can be detected only after at least 48 h, or even later, and the appearance of secondary oxidation compounds is sequential, with differences of many hours between them, and in very different concentrations [8,29]. These differences suggest that both oxidation pathways are very different.

(d) The fact that fatty acids have smaller oxidative stability than alkyl and glyceryl esters suggests that the structural units formed in the oxidation during in vitro digestion, shown in Table 2 and Figure 3, belong to fatty acids instead of to acyl groups. The immediate consequence is that these oxidized fatty acids are able to be absorbed. Their concentrations, given in Table 2 in mmol per mol of FA + AG in the digestate, coincide with their bioaccessibility. It should be commented on that the bioaccessibility of these oxidation compounds is very low, in the range of between 0.3–2.8 mmol per mol of FA + AG in the digestate. The total bioaccessibility of these kinds of compounds ranges from 1.5 mmol (in the sample enriched with the lowest concentration of *gamma*-tocopherol) to 8.9 mmol (in the sample enriched with the highest level of *alpha*-tocopherol). Nevertheless, taking into account that these oxidized fatty acids are toxic, their absorption has direct detrimental effects on human health [6,32,33,34]. By contrast, these compounds are not present in the digestates of the samples enriched with 2% and 5% of *gamma*-tocopherol, because in the digestion of these samples, no oxidation is produced.

(e) These results are in agreement with the degradation observed in the linoleic structures during digestion in the different samples (see Table 2). They also reinforce the differences (not statistically significant) found in the B_OMC_ values (see Table 1).

(f) From all the above mentioned, it can be deduced that an in-depth analysis, case by case, of the safety of enriching foods with compounds considered antioxidant should be mandatory, and caution should be taken in the indiscriminate intake of certain supplements. It would also be advisable to review the suitability of the European legislation that permits enrichment of refined edible oils with *alpha*-tocopherol under the principle of “*quantum satis*”.

#### 3.3.2. Information Provided by SPME-GC/MS about the Occurrence of Oxidation Reactions during In Vitro Digestion

The aim of this section is to analyse if the results obtained from ^1^H NMR data in previous sections are confirmed by the information provided by SPME-GC/MS. This technique allows us to estimate the abundance of volatile oxidation markers present in the headspace of the samples. Taking into account that the liquid matrix of the samples’ subject of study is similar, the abundance of the same volatile component in the different samples is valid for comparative purposes. The most common volatile oxidation markers formed in the oxidation of edible oils are well known. These are alkanals, (*E*)-2-alkenals, (*E,E*)-alkadienals, (*Z,E*)-alkadienals and oxygenated *alpha,beta* unsaturated aldehydes. Likewise, some furanones and furan derivatives are also well known oil oxidation markers [25].

Table 3 gives the abundances of the most important oxidation markers found in the headspace of the mixture made up of digestive juice, previously submitted the digestion conditions, and corn oil, in the same proportions as those employed in the digestion of the oil, this sample being considered as reference sample CDJ. Table 3 also gives the same information referring to the digestates of the corn oil DC, and of the corn oil samples enriched in both kinds of tocopherols, with a different enrichment degree (DC0.2γT, DC2γT, DC5γT and DC0.2αT, DC2αT, DC5αT). As an example, Figure S2 shows the region between 4–34 min of the total ion chromatogram obtained by SPME-GC/MS of the digestate of corn oil sample enriched in *alpha*-tocopherol DC5αT. In it, the peaks and retention times of some of the volatile oxidation compounds can be observed.

(1) Headspace of the mixture CDJ. It can be observed in Table 3 that, as expected, the headspace of the sample reference, CDJ, which contains the undigested corn oil, has the lowest number and concentration of oxidation markers of all samples. This is because the oil has not undergone digestion and as a consequence has not been oxidized. For this reason, its headspace only contains a reduced number of aldehydes, mainly alkanals, at a basal concentration, which is common in all non-oxidized edible oils.

(2) Corn oil digestate headspace. The headspace of the digestate DC contains a higher concentration of alkanals, of (*E*)-2-alkenals and of furan, 2-pentyl than CDJ and some 2,4-alkadienals, which are absent in the headspace of CDJ, all well known oxidation markers. The presence of these compounds in the headspace of DC proves that the corn oil has undergone oxidation during digestion.

(3)*Gamma*-tocopherol enriched corn oil samples headspace. The antioxidant effect exhibited by *gamma*-tocopherol can also be inferred by comparison of the headspace composition of DC0.2γT, DC2γT and DC5γT samples, with that of the headspace of the sample DC. In the headspace of the digestates of the three in *gamma*-tocopherol enriched samples, the same oxidation markers have been found as in DC but in a small abundance in all cases. This may be due to these three samples having undergone an oxidation process of a much smaller intensity than DC, resulting from the action of the added gamma-tocopherol. In agreement with the results obtained by ^1^H NMR, this effect is much more evident in the most gamma-tocopherol enriched samples (DC2γT and DC5γT), in which the abundance of (*E*)-2-alkenals is of a similar order to that found in CDJ.

(4) *Alpha*-tocopherol enriched corn oil samples headspace. The comparison of the headspaces’ composition of the digestates of the oil samples enriched in *alpha*-tocopherol DC0.2αT, DC2αT and DC5αT with that of the headspace of DC evidence, beyond a doubt, the prooxidant behaviour of this compound. The headspace of this digestate contains not only the oxidation compounds detected in DC in much more abundance, but also new oxidation markers, not present in DC, including alkanals, (*E*)-2-alkenals, 2,4-alkadienals and even oxygenated *alpha,beta* unsaturated aldehydes of known toxicity, furanones and a significant number of furan derivatives, some of these in considerable abundance. The great abundance of 2,4-alkadienals and furan, 2-pentyl and the presence of a large number of oxidation markers which absent in the headspace of DC, evidences the greater prooxidant role of *alpha*-tocopherol, in line with its higher concentration in the oil sample. In summary, the results obtained by studying the headspaces of the digestates are in total agreement with those obtained by ^1^H NMR.

In addition, a consideration can also be made, from the comparison between the ratio of the abundances of some of these oxidation markers generated under in *vitro* digestion conditions and under accelerated storage conditions. It is known that the oxidation of oils rich in linoleic acyl groups, such as corn oil, under accelerated storage conditions, form (*E,E*)-2,4-decadienals in much more abundance than (*Z,E*)-2,4-decadienals (near 4 times higher) [25,35]. However, under in vitro digestion conditions, both alkadienals are formed in similar abundance (see data of DC, DC0.2γT, DC2γT, DC5γT and DC0.2αT in Table 3), and in fact the ratio between the abundances of these two oxidation markers is reversed in the samples enriched with the higher concentrations of *alpha*-tocopherol (DC2αT and DC5αT). Moreover, the ratio between furan, 2-pentyl and 2,4-decadienals abundances is fairly different between oils submitted to accelerated storage conditions and those submitted to in vitro digestion. In the first case, this ratio for oils rich in linoleic acyl groups is always smaller than 1 [25,35]. However, in corn oil samples submitted to in vitro digestion, this ratio is always higher than 1, with the highest values in those samples with the lowest oxidation level (15.3 ± 2.3 in DC, 22.0 ± 0.1 in DC0.2γT, 27.7 ± 1.5 in DC2γT, 29.3 ± 2.7 in DC5γT). In fact, among all digestates, those coming from the samples enriched in *alpha*-tocopherol, which have the highest oxidation levels, exhibit the lowest values of this ratio (7.3 ± 2.3 in DC0.2αT, 5.3 ± 0.7 in DC2αT, and 4.6 ± 1.5 in DC5αT). This shows, in agreement with the previous comments regarding results obtained by ^1^H NMR, that different mechanisms are involved in these two oxidation processes. It should be taken into account that nothing happens randomly in oxidation processes, but rather that all reactions are governed by the interactions established between all the components of the sample. Whenever a process is carried out under the same conditions with samples of the same composition, the same results are obtained.

### 3.4. Bioaccessibility of Gamma- and Alpha-Tocopherols in the Different Digestates. Influence of the Enrichment with Alpha-Tocopherol on the Bioaccessibility of Gamma–Tocopherol Naturally Present in Corn Oil

The concentration of these tocopherols in the corn oil or in the digestates can be determined from the intensity of some of their ^1^H NMR spectral signals, as indicated in the experimental section, by using the equation [S 12], shown in Supplementary Material. This is possible because, as Table S5, Figure 2 and Figure S1 show, both tocopherols have some spectral signals (*gamma*-tocopherol a singlet at 6.36 ppm and *alpha*-tocopherol another singlet at 2.16 ppm), not overlapping with either that of the oil component signals, nor with those of the oxidation compounds formed during digestion.

In this case, data provided by ^1^H NMR spectroscopy allow one to estimate the bioaccessibility of these compounds, which can be expressed in two different ways, thus providing very complete information. One way of describing the bioaccessibility of tocopherols could be through the parameter B_T,_ defined by the ratio between the concentration of tocopherol (T) in the digestate (D) and the concentration of fatty acids plus acyl groups in the same sample (B_T_ = ([T_D_]/[FA + AG]_D_)). This parameter informs about the amount of tocopherol (given in mmol) that can be absorbed in relation to the amount of the main lipid components in the sample (given in mol). B_T_ allows us to contextualize the importance of the bioaccessibility of tocopherols, in quantitative terms, in the whole digestate. In addition, this parameter also allows comparison with the bioaccessibility of the other compounds before mentioned, which are also present in the digestates, such as the bioaccessible main components of the oil and the toxic compounds formed in the oxidation process.

Another way to express bioaccessibility, B´_T_, is through the ratio between the concentration of tocopherol in the digestate, T_D_ (given in mmol), and the concentration of tocopherol in the oil before digestion T_O_ (also given in mmol), (B´_T_ = ([T_D_]/[T_O_])). Parameter B´_T_ gives information about the amount of this compound lost during in vitro digestion and indicates the fraction of the original amount of this compound in the oil that, after digestion, is available to be absorbed. The bioaccessibility data obtained for the different samples, estimated using both approaches mentioned, are given in Table 4.

(1) Regarding the bioaccessibility of *gamma*-tocopherol naturally present in the corn oil C, the B_γ__T_ value of DC digestate (0.33 ± 0.00 mmol/mol (FA + AG)) indicates that a certain amount of this compound remains after in vitro digestion without degrading. This amount is very small in comparison with that of the toxic oxidation compounds also present in the same sample (see data in Table 2). Moreover, B´_γ__T_ is 0.67 ± 0.0 mmol/mmol(γT_O_)_,_ indicating that up to around 67% of the *gamma*-tocopherol contained in the oil remains in the digestate without degrading and is able to be absorbed. A similar value to this has been found with reference to *gamma*-tocopherol in the in vitro digestion of broccoli [36]. Both parameters, B_γ__T_ and B´_γ__T,_ also show that oxidation occurs during this in vitro digestion even having gamma-tocopherol, at this low concentration, without degrading. This is not surprising because oil oxidation and *gamma*-tocopherol degradation run in parallel in the oxidation of corn oil, under other conditions than these for a certain period of time, that is to say the presence of *gamma*-tocopherol does not avoid oil oxidation, but only slows down the process [35].

(2) Regarding the bioaccessibility of *gamma*-tocopherol in the digestates of the samples enriched with this compound. As Table 4 shows the B_γ__T_ values of the digestates are, as could be expected, higher as higher the enrichment degree of the sample is. These range between 0.89 ± 0.18 and 35.65 ± 1.12 mmol/mol (FA + AG). In sample DC0.2γT, in agreement with what takes place in the non-enriched sample, oxidation of the oil main components has been produced (see Table 2), although a certain amount of undegraded *gamma*-tocopherol remains in the sample. However, in the samples enriched with higher levels of *gamma*-tocopherol (DC2γT and DC5γT), this compound avoids the oxidation of corn oil main components during this in vitro digestion. The ratio between the amount of *gamma*-tocopherol that remain undegraded after digestion and the initial, given by B’_γ__T_, also shows high values (see Table 4), but in all cases a certain amount (between 35%–18%) of *gamma*-tocopherol has been lost due to its degradation, in this case avoiding totally or partially the oil component oxidation during in vitro digestion.

(3) Regarding the bioaccessibility of *gamma*-tocopherol in the digestates of the samples enriched with *alpha*-tocopherol. The B_γ__T_ values of these samples, as expected, are small because this compound is a minor corn oil component. They are given in Table 4. It is noteworthy that in the digestate of the less *alpha*-tocopherol enriched sample DC0.2αT, B_γ__T_ reaches the same value as in the digestate of the unenriched DC. This indicates that oil enrichment with this small amount of *alpha*-tocopherol has no effect on *gamma*-tocopherol degradation. Furthermore, in the other two enriched samples DC2αT and DC5αT, the B_γ__T_ value is almost the same for both samples (near 0.46 mmol/mol (FA + AG)). Bearing in mind that the concentration of *gamma*-tocopherol naturally present in corn oil C is 0.49 ± 0.00 mmol/mol (FA + AG), it is evident that this compound has almost undergone no degradation during in vitro digestion in the presence of high concentrations of *alpha*-tocopherol. As a result, B´_γ__T_ has the same value (0.67 ± 0.01) in sample DC0.2αT as in DC, and reaches 0.94–0.96 values in samples DC2αT and DC5αT. It is noteworthy that although *gamma*-tocopherol acts as an antioxidant, and alpha-tocopherol as a prooxidant, the concentration of *gamma*-tocopherol remains unaltered during the in vitro digestion in the presence of high concentrations of *alpha*-tocopherol. This could be due to the great difference in concentration between *gamma*- and *alpha*-tocopherol in these two latter samples. This leads one to think that this great difference in concentration significantly reduces the probability of *gamma*-tocopherol molecules being near the oxidation sites of the oil main components, in comparison with that of *alpha*-tocopherol. This could explain *gamma*-tocopherol concentration remaining unaltered during digestion in these samples.

(4) Regarding the bioaccessibility of *alpha*-tocopherol in the digestates of the samples enriched with this compound. The bioaccesibility of *alpha*-tocopherol is null or small depending on the enrichment degree. B_αT_ data given in Table 4 indicate that the added *alpha*-tocopherol degrade during digestion to a greater extent than the added *gamma*-tocopherol. In fact, *alpha*-tocopherol totally degrades in the sample having the smaller enrichment level. For this reason, B_αT_ and B´_αT_ values are zero in the less enriched sample. In addition, in the other two samples (DC2αT and DC5αT) B´_αT_ shows small values, especially in the most enriched sample. These results are in agreement with others recently published [37]. The participation of this compound in the oxidative reactions taking place during in vitro digestion could explain their high level of degradation.

## 4. Conclusions

The lipolysis extent reached in the in vitro digestion of corn oil and of samples enriched in *gamma*- and *alpha*-tocopherol is high and of a similar order in all cases. The effect of the enrichment with these compounds does not provokes significant changes in this process, even if the results could suggest a slightly increase in lipolysis extent in the case of enrichment with *gamma*-tocopherol and a slight decrease in the case of *alpha*-tocopherol. These subtle differences could be attributed to the small differences in polarity and in other molecular characteristics of both tocopherols. The lipolysis pattern is also very similar in all samples, glycerol and mono-glycerides being the main glyceride structures present in the digestates, followed by triglycerides. However, diglycerides are the structures of which abundance is the smallest. This hydrolytic pattern could be considered typical of the enzymatic cocktail and digestive juices used. As a consequence of the high lipolysis extent and of its pattern, high bioaccessibility is reached for oil main components, namely fatty acids and monoglycerides. This ranges between 0.64 and 0.72 mol ([FA] + [MG])_D_/mol([FA] + [AG])_D_. The subtle differences among samples, regarding the lipolysis extent, are also translated to bioaccesibility of oil main components: the highest value is exhibited by the sample most enriched with *gamma*-tocopherol, and the opposite is true for the sample most enriched with *alpha*-tocopherol.

In agreement with several previous studies, it is again confirmed that in vitro digestion provokes lipid oxidation. This is confirmed by the clear diminution of linoleic structures in the digestate in relation to those existing in the oil, which prove their degradation during in vitro digestion. The analysis of the concentration of these linoleic structures in the digestates of the oil samples enriched in *gamma*- and in *alpha*-tocopherol indicates that the former tocopherol avoids, to a certain degree, the degradation of linoleic, acting as an antioxidant, whereas the opposite is true for the latter compound. It has been proved that enrichment of the oil with *gamma*-tocopherol at a level of 2% in weight or higher avoids oil component oxidation during in vitro digestion. However, an enrichment level of 0.2 in the weight of *gamma*-tocopherol, barely brings about a reduction in oxidation degree. From these results, it is evident that this compound behaves as an antioxidant. However, *alpha*-tocopherol behaves as a prooxidant, in line with its higher enrichment degree. Oxidation under in vitro digestion conditions evolves at a very high rate, generating very different oxidation compounds in concentrations of a similar order, unlike what happens in other oxidation processes. At high enrichment degrees of *alpha*-tocopherol, hydroperoxy-, hydroxy- and keto-conjugated dienes as well as keto-*E*-epoxy-*E*-monoenes and aldehydes have been detected in the digestates, all of them well known oxidation markers, and some of them associated to degenerative diseases. The study of the volatile components of the different digestates leads to the same conclusions regarding the behaviour of *gamma*-tocopherol as an antioxidant and of *alpha*-tocopherol as a prooxidant. Once again, the usefulness of the two techniques employed in this study is proven, as well as the need to use as many oxidation markers as possible, in order to have the most complete picture as possible of the oxidation process, and in order to avoid erroneous interpretations.

The bioaccessibility of the naturally present *gamma*-tocopherol in the corn oil cannot be considered low, although a certain amount of this compound has been lost during in vitro digestion. Nevertheless, this bioaccessibility is smaller than that of the toxic oxidation compounds generated during in vitro digestion. *Gamma*-tocopherol bioaccessibility in the samples enriched in this compound is higher, when the enrichment level is higher, showing high values, in addition to avoiding the oxidation of oil components during in vitro digestion. The bioaccessibility of the naturally present *gamma*-tocopherol in the oil samples enriched in *alpha*-tocopherol is nearly one hundred percent in the most enriched samples, in spite of *alpha*-tocopherol provoking oil component oxidation in line with its higher enrichment degree. By contrast, alpha-tocopherol exhibits a low bioaccessibility, being even null in the less enriched sample, probably due to its behaviour as a prooxidant. Thus, the safety of the intake of supplements which are rich in *alpha*-tocopherol should be the subject of broader and deeper studies. Likewise, the suitability of European legislation that allows enriching edible oils with this compound under the principle of “*quantum satis*” should be reviewed.

## Figures and Tables

**Table 1:** Lipolysis extent. Molar percentages of triglycerides (TG), diglycerides (1,2-DG and 1,3-DG), monoglycerides (2-MG and 1-MG) and glycerol (Gol), in relation to the total glyceride structures, in corn oil C, in the digestates of this oil DC and in those of the samples enriched in *gamma*- and *alph*a-tocopherol (DC0.2γT, DC2γT, DC5γT DC0.2αT, DC2αT and DC5αT). in vitro bioaccessibility of oil main components B_OMC_, defined by the ratio (mol [FA]+[MG])_D_/mol ([FA]+[AG])_D_. Different letter within each column indicate statistically significant difference among the samples (*p* < 0.05).

Sample	Lipolysis Extent						
	TG (%)	1,2-DG (%)	1,3-DG (%)	2-MG (%)	1-MG (%)	Gol (%)	B_OMC_
C	99.8 ±0.2^a^	1.1±0.1^a^	-	-	-	-	-
DC	22.3±5.9^b^	14.0±1.6^b^	1.8±1.0^a^	26.6±5.6^a^	4.4±1.1^a^	30.8±1.8^a^	0.67±0.07^a^
DC0.2γT	22.2±4.4^b^	14.7±1.0^b^	1.7±0.2^a^	26.0±1.6^a^	4.1±0.7^a^	31.3±1.6^a^	0.67±0.04^a^
DC2γT	20.7±3.4^b^	14.1±2.2^b^	1.4±0.5^a^	28.2±2.7^a^	5.3±0.4^a^	30.3±2.6^a^	0.69±0.02^a^
DC5γT	17.0±7.8^b^	14.3±0.6^b^	1.7±0.9^a^	30.2±6.0^a^	5.1±2.3^a^	31.7±0.2^a^	0.72±0.08^a^
DC0.2αT	20.1±4.3^b^	13.9±0.7^b^	1.5±0.5^a^	28.0±2.9^a^	5.0±0.5^a^	31.4±1.9^a^	0.70±0.05^a^
DC2αT	21.8±1.8^b^	14.1±1.1^b^	1.9±0.2^a^	28.5±2.9^a^	5.3±0.4^a^	28.5±0.6^a^	0.68±0.02^a^
DC5αT	24.8±2.0^b^	14.9±0.9^b^	1.5±0.7^a^	24.7±2.9^a^	4.3±0.2^a^	29.9±1.1^a^	0.64±0.03^a^

Different letter within each column indicate statistically significant difference among the samples (*p* < 0.05). This is already defined in the heading of the table; -: not detected.

**Table 2:** Molar percentage of linoleic fatty acids (FA) plus acyl groups (AG) (FA+AG), in relation to the total moles of all kinds of AG and FA, in the corn oil C, in the digestates of this oil DC, and in those of the oil samples enriched in *gamma*- and *alpha*-tocopherol (DC0.2γT, DC2γT, DC5γT, DC0.2αT, DC2αT and DC5αT). Concentration of some oxidation compounds, expressed by mmol per mol of AG+FA in the above samples. Different letters within each column indicate statistically significant difference among the samples (*p* < 0.05).

Samples	Linoleic FA+AG (%)	Concentration of Some Oxidation Compounds (mmol/mol(AG + FA))					
		HPO-c(*Z,E*)dEs	HOc(*Z,E*) dEs	HO-c(*E,E*) dEs	KO-c(*Z,E*) dEs	KO-*E*-EPO-*E*-mEs	n- Alkanals
C	49.2±0.5^a^	-	-	-	-	-	-
DC	41.3±0.0^b^	1.8 ±0.31^a^	-	-	-	-	-
DC0.2γT	43.3± 0.0^c^	1.54±0.27^a^	-	-	-	-	-
DC2γT	43.1±0.2^c^	-	-	-	-	-	-
DC5γT	44.4± 0.3^c^	-	-	-	-	-	-
DC0.2αT	40.5±0.6^b^	2.77±0.25^b^	-	-	-	-	-
DC2αT	40.8± 0.0^b^	2.21±0.31^a,b^	1.22±0.07^a^	2.27±0.00^a^	0.53±0.03^a^	0.71±0.00^a^	-
DC5αT	40.6±1.2^b^	2.06±0.38^a,b^	2.78±0.19^b^	2.43±0.71^a^	1.09±0.14^b^	1.12±0.16^b^	0.27±0.09

-: not detected; HPO-c(*Z,E*)dEs: hydroperoxy-conjugated-*(Z,E*)-dienes; HO-c(*Z,E*)dEs: hydroxy-conjugated-(*Z,E*)-dienes; HO-c(*E,E*)dEs: hydroxy-conjugated-(*E,E*)-dienes; KO-c(*Z,E*)dEs: keto-conjugated-(*Z,E*)-dienes; KO-*E*-EPO-*E*-mEs: keto-(*E*)-epoxy-(*E*)-monoenes. Different letter within each column indicate statistically significant difference among the samples (*p* < 0.05). This is already defined in the heading of the table.

**Table 3:** Abundances of some aldehydes (alkanals, (*E*)-2-alkenals, 2,4-alkadienals and oxygenated *alpha,beta* unsaturated aldehydes), furanones and furan derivatives identified by SPME-GC/MS, in the headspace of the mixture of digestive juices and non-enriched corn oil sample (CDJ), in the digestates of the corn oil (DC) and in those of oil samples enriched in *gamma*- and *alpha*-tocopherol (DC0.2γT, DC2γT, DC5γT DC0.2αT, DC2αT and DC5αT). Data are given in area counts of their mass spectra base peak (Bp) multiplied by 10^-6^, and obtained as an average of two determinations, together with their standard deviations.

Compound (Molecular Weight)	Bp	CDJ	DC	DC0.2γT	DC2γT	DC5γT	DC0.2αT	DC2αT	DC5αT
Aldehydes									
**Alkanals**									
Pentanal (86)*	44	17.8 ± 0.9	41.4 ± 1.8	36.0 ± 0.5	48.4 ± 2.9	22.3 ± 1.8	41.4 ± 5.4	52.4 ± 7.3	83.9 ± 6.4
Hexanal (100)*	44	12.9 ± 3.2	76.0 ± 3.1	79.1 ± 11.8	72.4 ± 4.9	45.6 ± 4.9	184.4 ± 12.3	480.6 ± 75.3	604.0 ± 56.0
Heptanal (114)*	70	0.7 ± 0.0	3.0 ± 1.2	4.4 ± 0.7	3.5 ± 0.5	2.4 ± 0.4	6.8 ± 0.1	51.2 ± 11.7	164.3 ± 2.1
Octanal (128)*	41	-	-	-	-	-	-	34.0 ± 6.4	46.4 ± 5.1
Nonanal (142)*	57	3.3 ± 0.2	11.4 ± 0.3	11.3 ± 0.5	17.0 ± 1.7	17.1 ± 1.1	12.4 ± 1.2	42.6 ± 2.4	69.7 ± 11.4
Decanal (156)*	41	-	-	-	-	-	-	-	4.6 ± 0.5
**Total**		**34.7 ± 2.1**	**131.8 ± 0.1**	**130.8 ± 12.47**	**141.3 ± 10.1**	**87.5 ± 8.3**	**245.1 ± 5.9**	**660.8 ± 103.1**	**972.9 ± 64.4**
**(E)-2-alkenals**									
(*E*)-2-Butenal (70)*	70	19.5 ± 2.8	20.6 ± 3.4	20.8 ± 5.8	16.1 ± 2.3	15.2 ± 2.6	18.2 ± 3.3	20.7 ± 2.3	23.0± 7.8
(*E*)-2-Pentenal (84)	55	-	-	-	-	-	-	1.3 ± 0.0	5.1 ± 1.1
(*Z*)-4-Heptenal (112)	41	-	-	-	-	-	-	3.9 ± 0.7	5.4 ± 0.5
(*E*)-2-Heptenal (112)*	41	2.3 ± 0.3	47.4 ± 8.1	40.2 ± 6.4	16.5 ± 8.9	8.8 ± 3.0	64.5 ± 8.7	186.3 ± 28.3	275.0 ± 47.7
(*E*)-2-Octenal (126)*	70	-	-	-	-	-	42.3 ± 2.2	186.3 ± 26.2	346.3 ± 58.9
(*E*)-2-Nonenal (140)*	55	-	1.2 ± 0.2	1.1 ± 0.1	1.4 ± 0.1	1.8 ± 0.1	1.3 ± 0.1	2.8 ± 0.8	4.7 ± 0.4
(*E*)-2-Decenal (154)*	70	-	-	-	-	-	4.1 ± 0.6	6.7 ± 1.0	10.8 ± 1.8
(*E*)-2-Undecenal (168)*	70	-	-	-	-	-	0.2 ± 0.0	1.4 ± 0.3	3.0 ± 0.4
**Total**		**21.8 ± 3.1**	**69.2 ± 4.9**	**62.08 ± 12.28**	**38.9 ± 4.4**	**25.8 ± 5.4**	**130.6 ± 8.3**	**409.3 ± 58.1**	**673.3 ± 102.0**
**2,4-Alkadienals**									
(*Z,E*)-2,4-Heptadienal (110)	81	-	-	-	-	-	3.8 ± 0.1	24.0 ± 4.9	38.6 ± 1.6
(*E,E*)-2,4-Heptadienal (110)*	81	-	-	-	-	-	3.5 ± 0.5	24.4 ± 3.0	36.4 ± 10.5
(*Z,E*)-2,4-Octadienal (124)	81	-	-	-	-	-	-	-	1.9 ± 1.0
(*E,E*)-2,4-Octadienal (124)	81	-	-	-	-	-	0.8 ± 0.0	3.4 ± 0.1	6.1 ± 0.8
(*E,E*)-2,4-Nonadienal (138)	81	-	1.0 ± 0.1	0.7 ± 0.2	0.7 ± 0.1	0.4 ± 0.1	1.2 ± 0.2	1.9 ± 0.2	2.4 ± 0.5
(*Z,E*)-2,4-Decadienal (152)	81	-	0.6 ± 0.0	0.4 ± 0.0	0.4 ± 0.1	0.3 ± 0.0	2.6 ± 0.8	19.3 ± 3.1	38.4 ± 10.1
(*E,E*)-2,4-Decadienal (152)*	81	-	0.8 ± 0.1	0.5 ± 0.0	0.3 ± 0.1	0.3 ± 0.1	3.3 ± 0.3	15.8 ± 0.5	29.0 ± 13.2
**Total**		**-**	**2.4 ± 0.2**	**1.6 ± 0.3**	**1.3 ± 0.1**	**1.0 ± 0.2**	**15.3 ± 1.6**	**88.7 ± 11.7**	**152.8 ± 37.6**
**Oxigenated α,β-unsaturated**									
4,5-epoxy-(*E*)-2-heptenal (126)	68	-	-	-	-	-	-	0.9 ± 0.2	3.1 ± 1.0
4,5-epoxy-2-decenal (isomer) (168)	68	-	-	-	-	-	0.4 ± 0.1	2.7 ± 0.2	4.3 ± 2.2
4,5-epoxy-(*E*)-2-decenal (168)*	68	-	-	-	-	-	1.1 ± 0.2	10.9 ± 0.4	31.8 ± 0.0
**Total**		**-**	**-**	**-**	**-**	**-**	**1.5 ± 0.3**	**15.0 ± 0.1**	**41.1 ± 3.2**
**Furanones**									
5-butyl-5H-furan-2-one (140)	84	-	-	-	-	-	-	0.9 ± 0.0	1.6 ± 0.1
5-pentyl-2(3H)-furanone (154) (or isomer)	98	-	-	-	-	-	-	0.5± 0.0	0.9± 0.2
5-pentyl-2(5H)-furanone (154)	84	-	-	-	-	-	-	2.7 ± 0.3	4.4 ± 0.9
**Total**		**-**	**-**	**-**	**-**	**-**	**-**	**4.1 ± 0.4**	**6.9** **± 1.2**
**Furan derivatives**									
Furan, 2-ethyl (96)	81	-	-	-	-	-	0.2 ± 0.1	0.3 ± 0.1	0.6 ± 0.0
Furan, 2-propyl (110)	81	-	-	-	-	-	0.2 ± 0.0	0.6 ± 0.0	0.9 ± 0.1
Furan, 2-butyl (124)	81	-	0.5 ± 0.0	0.6 ± 0.2	0.6 ± 0.1	0.5 ± 0.1	0.6 ± 0.0	2.5 ± 0.5	5.4 ± 0.1
Furan, 2-pentyl (138)*	81	4.7 ± 1.4	21.5 ± 1.5	19.8 ± 0.8	19.4 ± 0.5	17.6 ± 1.1	43.5 ± 2.1	186.8 ± 42.8	308.3 ± 21.4
Furan, 2-heptyl (166)	81	-	-	-	-	-	-	0.7 ± 0.2	8.3 ± 1.7
**Total**		**4.7 ± 1.4**	**22.4 ± 1.5**	**20.4 ± 0.6**	**20.1 ± 0.4**	**18.1 ± 1.0**	**44.5 ± 2.2**	**190.8 ± 43.5**	**316.0 ± 21.5**

*Asterisked compounds were acquired commercially and used as standards for identification purposes; -: not detected.

**Table 4:** Bioaccessibility of *gamma-*tocopherol (γT) and *alpha*-tocopherol (αT) in the digestates of the different samples, in which these compounds are present, expressed in two different ways. B_T =_ (mmol T_D_/mol (AF+GA) _D_) and B’_T =_ (mmol T_D_/mmol T_O_). Values are the average of two determinations, together with their standard deviations.

Samples	B_γT_	B´_γ__T_	B_αT_	B´_α__T_
DC	0.33 ± 0.00	0.67 ± 0.00		
DC0.2γT	0.89 ± 0.18	0.65 ± 0.14		
DC2γT	8.55 ± 0.00	0.70 ± 0.01		
DC5γT	35.65 ± 1.12	0.82 ± 0.03		
DC0.2αT	0.33 ± 0.03	0.67 ± 0.01	-	-
DC2αT	0.46 ± 0.02	0.94 ± 0.00	6.36 ± 0.00	0.58 ± 0.00
DC5αT	0.47 ± 0.04	0.96 ± 0.01	12.91 ± 0.15	0.32 ± 0.00

-: not detected.

## Supplementary Materials

The following are available online at https://www.mdpi.com/2076-3921/9/3/246/s1, Table S1: Composition and pH values of the juices employed in the in vitro digestion model employed in this study; Table S2: Chemical shift assignments and multiplicities of the ^1^H NMR signals in CDCl_3_ of protons of glycerides. TG: triglycerides; DG: diglycerides; MG: monoglycerides. The signal letters agree with those given in Figure 1; Table S3: Chemical shift assignments and multiplicities of the ^1^H NMR signals in CDCl_3_ of protons of acyl groups and fatty acids. AG: acyl groups; FA: fatty acids. The signal letters agree with those given in Figure 1; Table S4: Chemical shift assignments and multiplicities of the ^1^H NMR signals in CDCl_3_ of protons of some oxidation compounds detected in the digestates and formed during the in vitro digestion. The signal letters agree with those given in Figure 2; Table S5: Chemical shift assignments and multiplicities of the ^1^H NMR signals in CDCl_3_ of protons of *gamma*- and *alpha*-tocopherol present in the samples before and after in vitro digestion. The signal letters agree with those given in Figure 2; Figure S1: Chemical structures of tocopherols involved in this of study, together with some chemical shifts (ppm) of some of their hydrogen atoms. Equations used for the quantification from ^1^H NMR spectral data of several compounds present in the starting samples and/or in the lipid extracts of the digestates. Figure S2: Region between 4-34 min of the total ion chromatogram obtained by SPME-GC/MS of the digestate of corn oil sample enriched in *alpha*-tocopherol DC5αT. Peaks identified: **(1)** pentanal; **(2)** hexanal; **(3)** furan, 2-butyl; **(4)** heptanal; **(5)** (*E*)-2-heptenal; **(6)** furan, 2-pentyl; **(7)** (*Z,E*)-2,4-heptadienal; **(8)** (*E,E*)-2,4-heptadienal; **(9)** (*E*)-2-octenal; **(10)** nonanal; **(11)** (*E*)-2-nonenal; **(12)** decanal; **(13)** (*E*)-2-decenal; **(14)** (*Z,E*)-2,4-decadienal; **(15)** (*E,E*)-2,4-decadienal; **(16)** 5-pentyl-2(5H)-furanone; **(17)** 4,5-epoxy-2-decenal (isomer); **(18)** 4,5-epoxy-(*E*)-2-decenal.
