# Supplementary material for: Effect of the Enrichment of Corn Oil With alpha- or gamma-Tocopherol on Its In Vitro Digestion Studied by 1H NMR and SPME-GC/MS; Formation of Hydroperoxy-, Hydroxy-, Keto-Dienes and Keto-E-epoxy-E-Monoenes in the More alpha-Tocopherol Enriched Samples
Source: Antioxidants (Basel). 2020 Mar 18;9(3):246. doi: 10.3390/antiox9030246 (PMC7139825; doi:10.3390/antiox9030246)
Supplement: Supplementary file 1 [file antioxidants-09-00246-s001.pdf]

SUPPLEMANTARY MATERIAL

Effect of the enrichment of corn oil with *alpha-* or *gamma*-tocopherol on its *in vitro* digestion studied by ^{1}H NMR and SPME-GC/MS. Formation of hydroperoxy-, hydroxy-, keto-dienes and keto-*E*-epoxy-*E*-monoenes in the more *alpha*-tocopherol enriched samples

J. Alberdi-Cedeño, María L. Ibargoitia, María D. Guillén*

Food Technology. Faculty of Pharmacy. Lascaray Research Center. University of the Basque Country (UPV-EHU). Paseo de la Universidad nº7, 01006 Vitoria-Gasteiz, Spain, Telf: 34-945-013081, Fax: 34-945-013014.

E-mail: mariadolores.guillen@ehu.es*

Table S1. Composition and pH values of the juices employed in the *in vitro* digestion model employed in this study.

**Components**	**Saliva**	**Gastric juice**	**Duodenal juice**	**Bile juice**
KCl (mmol/L)	12.02	11.06	7.57	5.05
NaCl (mmol/L)	5.10	47.09	119.98	89.99
$NaHCO_3$ (mmol/L)	20.17	-	40.33	68.86
NaH_2PO_4 (mmol/L)	7.40	0.22	-	-
NH_4Cl (mmol/L)	-	5.72	-	-
KH_2PO_4 (mmol/L)	-	-	0.59	-
Na_2SO_4 (mmol/L)	4.79	-	-	-
KSCN (mmol/L)	2.06	-	-	-
$MgCl_2$ (mmol/L)	-	-	0.53	-
$CaCl_2$*$2H_2O$ (mmol/L)	-	2.72	1.36	1.51
HCl (37%) (mL/L)	-	6.50	0.18	0.15
Urea (mmol/L)	3.33	1.42	1.67	4.16
Glucose (mmol/L)	-	3.61	-	-
Glucuronic acid (mmol/L)	-	0.10	-	-
Uric acid (mmol/L)	0.09	-	-	-
Glucoseamine hydrochloride (mmol/L)	-	1.53	-	-
Bovine serum albumin (g/L)	-	1.00	1.00	1.80
Mucin (g/L)	0.025	3.00	-	-
A. oryzae α-amylase (g/L)	0.29	-	-	-
A. niger lipase (U/mL)	-	100	-	-
Pepsin (g/L)	-	2.50	-	-
Pancreatin (g/L)	-	-	9.00	-
Lipase type II from porcine pancreas (g/L)	-	-	1.50	-
Bovine bile extract (g/L)	-	-	-	18.75
**pH**	6.9 ±0.0	1.3 ±0.1	8.1 ±0.1	8.2 ±0.1

Table S2. Chemical shift assignments and multiplicities of the ^{1}H NMR signals in $CDCl_3$ of protons of glycerides. TG: triglycerides; DG: diglycerides; MG: monoglycerides. The signal letters agree with those given in Figure 1.

Signal	Chemical shift (ppm)	Multiplicity	Type of protons	Structures
			**Glycerides structure protons**	
**I**	3.65	ddd	ROCH$_2$–CHOH–C**H$_2$**OH	glyceryl group in **1-MG**
**J**	3.73	m*	ROCH$_2$–CH(O*R*')–C**H$_2$**OH	glyceryl group in **1,2-DG**
**K**	3.84	m*	HOC**H$_2$**–CH(O*R*)–C**H$_2$**OH	glyceryl group in **2-MG**
**L**	3.94	m	ROCH$_2$–C**H**OH–CH$_2$OH	glyceryl group in **1-MG**
**M**	4.05–4.21	m	ROC**H$_2$**–C**H**OH–C**H$_2$**OR'	glyceryl group in **1,3-DG**
**N**	4.18	ddd	ROC**H$_2$**–CHOH–CH$_2$OH	glyceryl group in **1-MG**
**O**	4.22	dd,dd	ROC**H$_2$**–CH(O*R*')–C**H$_2$**OR''	glyceryl group in **TG**
**P**	4.28	ddd	ROC**H$_2$**–CH(O*R*')–CH$_2$OH	glyceryl group in **1,2-DG**
**Q**	4.93	m	HOCH$_2$–C**H**(O*R*)–CH$_2$OH	glyceryl group in **2-MG**
**R**	5.08	m	ROCH$_2$–C**H**(O*R*')–CH$_2$OH	glyceryl group in **1,2-DG**

Abbreviations: d: doublet; m: multiplet.

*This signal shows different multiplicity if the spectrum, is acquired from the pure compound or taking part in the mixture.

**The intensity of some of these signals, also shown in Figure 1, together with signal F of Table S3, were used to estimate the molar percentages of different kinds of glyceryl structures using the equations [eq. S1-eq. S10].

***The assignment of the ^{1}H NMR signals of the protons was made as in previous studies (Guillén & Uriarte, 2012; Nieva-Echevarría et al., 2014).

Table S3. Chemical shift assignments and multiplicities of the ^{1}H NMR signals in $CDCl_3$ of protons of acyl groups and fatty acids. AG: acyl groups; FA: fatty acids. The signal letters agree with those given in Figure 1.

Signal	Chemical shift (ppm)	Multiplicity	Type of protons	Structures
			**Main acyl groups (AG) and fatty acids (FA)**	
A	0.88	t	$–C\underline{\mathbf{H}}_3$	saturated and monounsaturated **ω-9** in **AG** and **FA**
	0.89	t	$–C\underline{\mathbf{H}}_3$	linoleic in **AG** and **FA**
B	0.97	t	$–C\underline{\mathbf{H}}_3$	linolenic in **AG** and **FA**
C	1.19–1.42	m**	$–(C\underline{\mathbf{H}}_2)_n–$	**AG** and **FA**
D	1.61	m	$–OCO–CH_2–C\underline{\mathbf{H}}_2–$	**AG** in **TG**
	1.62	m	$–OCO–CH_2–C\underline{\mathbf{H}}_2–$	**AG** in **1,2-DG**
	1.63	m	$–OCO–CH_2–C\underline{\mathbf{H}}_2–$, $COOH–CH_2–C\underline{\mathbf{H}}_2–$	**AG** in **1,3-DG**, **1-MG** and **FA**
	1.64	m	$–OCO–CH_2–C\underline{\mathbf{H}}_2–$	**AG** in **2-MG**
E	1.92–2.15	m***	$–C\underline{\mathbf{H}}_2–CH=CH–$	**AG** and **FA**
F*	2.26–2.36	dt	$–OCO–C\underline{\mathbf{H}}_2–$	**AG** in **TG**
	2.33	m	$–OCO–C\underline{\mathbf{H}}_2–$	**AG** in **1,2-DG**
	2.35	t	$–OCO–C\underline{\mathbf{H}}_2–$, $COOH–C\underline{\mathbf{H}}_2–$	**AG** in **1,3-DG**, **1-MG** and **FA**
	2.38	t	$–OCO–C\underline{\mathbf{H}}_2–$	**AG** in **2-MG**
G*	2.77	t	$=HC–C\underline{\mathbf{H}}_2–CH=$	linoleic in **AG** and **FA**

Abbreviations: d: doublet; t: triplet; m: multiplet.
*The intensity of these signals, also shown in Figure 1, was used to estimate the molar percentage of linoleic structures by using equation [eq. S11].
**Overlapping of multiplets of methylenic protons in the different acyl groups either in *β*-position, or further, in relation to double bonds, or in *γ*-position, or further, in relation to the carbonyl group.
***Overlapping of multiplets of the α-methylenic protons in relation to a single double bond of the different unsaturated acyl groups.
****The assignment of the ^{1}H NMR signals of the protons was made as in previous studies (Guillén & Ruiz, 2003; Nieva-Echevarría et al., 2014).

Table S4. Chemical shift assignments and multiplicities of the ^{1}H NMR signals in $CDCl_3$ of protons of some oxidation compounds detected in the digestates and formed during the *in vitro* digestion. The signal letters agree with those given in Figure 2.

Signal	Chemical shift (ppm)	Multiplicity	Type of protons	Structures
			**Oxidation Compounds (OC)**	
**Conjugated dienic systems associated with hydroperoxy groups**				
**-**	5.51	dtm	–C**H**=CH–CH=CH–	(*Z,E*)-conjugated double bonds associated with hydroperoxy group (OOH) in octadecadienoic **AG** and **FA** **HPO-c(*Z,E*)dEs**
**-**	5.56	ddm		
**-**	6.00	ddtd		
**b**	***6.58***	dddd		
**Conjugated dienic systems associated with hydroxy groups**				
**–**	5.44	ddd	–C**H**=CH–CH=CH–	(*Z,E*)-conjugated double bonds associated with hydroxy group (OH) in octadecadienoic **AG** and **FA** **HO-c(*Z,E*)dEs**
**-**	5.66	dd		
**-**	5.97	t		
**a**	***6.49***	dd		
**-**	5.58	dd	–C**H**=CH–CH=CH–	*(E,E*)-conjugated double bonds associated with hydroxy group (OH) in octadecadienoic **AG** and **FA** **HO-c(*E,E*)dEs**
**-**	5.71	dd		
**-**	6.03	dd		
**c**	***6.18***	dd		
**Conjugated keto-dienes (KO-c-dEs)**				
**d**	***7.49***	ddd	-CH=C**H-** (C-11)	*(Z,E)*- conjugated double bonds associated with ketodiene of octadeadienoic **AG** and **FA**acyl groups **KO-c(*Z,E*)dEs**
	6.16	d	-CH=CH- (C-10)	
	6.12	m	-CH=CH- (C-12)	
	5.91	dt	-CH=CH- (C-13)	
	2.54	t	$-CH_2-CO$	
**Keto-epoxy-monoenes (KO-EPO-mES)**				
**e**	6.52	dd	-CH=CH**-**	
	6.38	d	-CH=CH-	13-KO-9,10-*E*-EPO-11-*E*-ene
	3.22	dd	-HCOC**H**-	9-KO-12,13-*E*-EPO-10-*E*-ene
	2.91	td	-HCOCH-	**KO-*E*-EPO-*E*-mEs**
	2.53	t	$-CH_2-$	
**Aldehydes**				
**f**	***9.75***	t	-C**H**O	**n-alkanals**
	2.40	dt	$-CH_2-$	

Abbreviations: d: doublet; t: triplet; m: multiplet.

*The intensities of the signals indicated in bold, together with signal F of Table S3, were used to estimate the concentration (mmol/molAG+FA) using the equation [eq. S12].

**The assignment of the ^{1}H NMR signals of the protons was made with the aid of standard compounds and with the data taken from literature (Guillén & Ruiz, 2005; Dufour & Loonis, 2005; Guillén & Goicoechea, 2007; Lin et al., 2007; Ramsden et al., 2017).

Table S5. Chemical shift assignments and multiplicities of the ^{1}H NMR signals in $CDCl_3$ of protons of some tocopherols detected in the samples before and after *in vitro* digestion. The signal letters agree with those given in Figure 2.

Signal	Chemical shift (ppm)	Multiplicity	Type of protons	Compounds
		**Tocopherols**		
**αT**	2.16**	s	-C**H₃** (C-7)	*alpha*-Tocopherol
**γT**	6.36**	s	-C**H** (C-5)	*gamma*-Tocopherol

Abbreviation: s: singlet.

*The intensity of these signals, together with signal F of Table S3, were used to estimate the concentration (mmol/molAG+FA) using the equation [eq. S12].

**Assignment was made with the aid of standard compounds and with the data taken from the literature (Baker & Mayers, 1991)

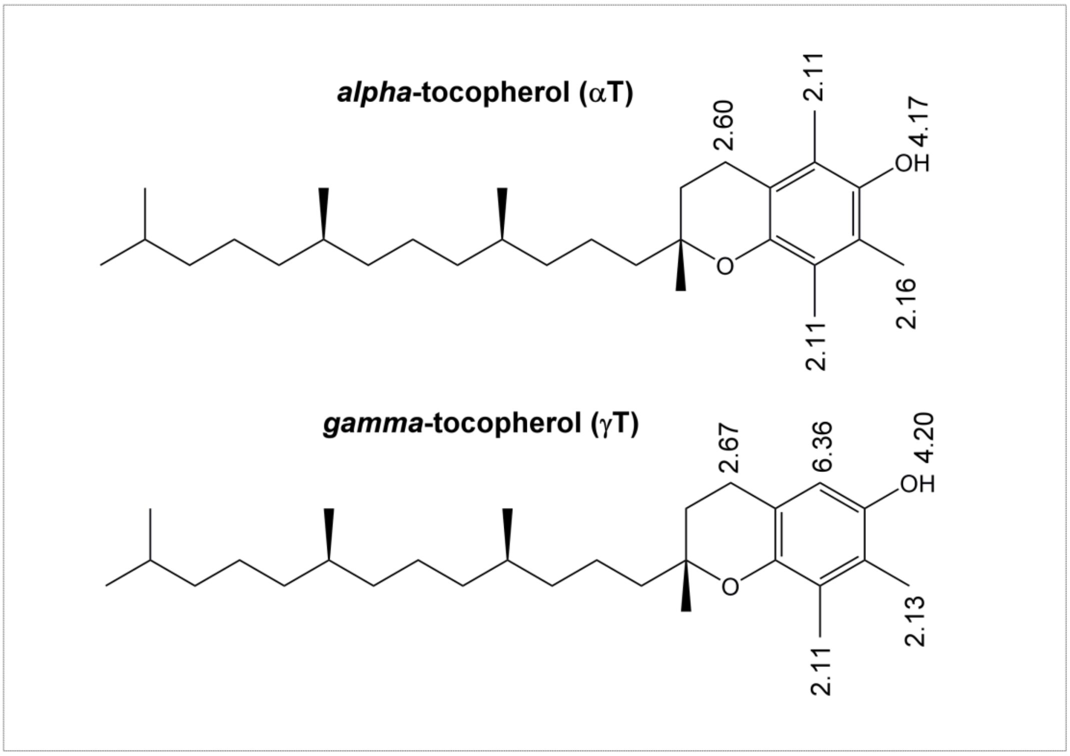

Figure S1. Chemical structures of tocopherols involved in this of study, together with some chemical shifts (ppm) of some of their hydrogen atoms.

Quantification from ^{1}H NMR spectral data of several compounds present in the starting samples and/or in the lipid extracts of the digestates

Bearing in mind that the area of each ^{1}H NMR spectral signal is proportional to the number of protons that generate it, and that the proportionality constant is the same for all kinds of protons, the area of some spectral signals can be employed to quantify a wide variety of compounds, as detailed below.

A. Equations used to estimate the molar percentage (%) of the several glyceride structures present in the lipid extract of digestates and the glycerol.

In these equations the number of moles (N) of fatty acids and all the glycerides were expressed as follows:

$N_{2\text{-}MG}=Pc*A_K/4$ [eq. S1]

$N_{1\text{-}MG}=Pc*A_L$ [eq. S2]

$N_{1,2\text{-}DG}=Pc*(A_{I+J}-2A_L)/2$ [eq. S3]

$N_{TG}=Pc*(2A_{4.26\text{-}4.38}-A_{I+J}+2A_L)/4$ [eq. S4]

$N_{1,3\text{-}DG}=Pc*(A_{4.04\text{-}4.38}-2A_{4.26\text{-}4.38}-2A_L)/5$ [eq. S5]

$N_{FA}=(Pc*A_F-6N_{TG}-4N_{1,2\text{-}DG}-4N_{1,3\text{-}DG}-2N_{1\text{-}MG}-2N_{2\text{-}MG})/2$ [eq. S6]

$N_{Gol}=(N_{FA}-N_{1,2\text{-}DG}-N_{1,3\text{-}DG}-2N_{2\text{-}MG}-2N_{1\text{-}MG})/3$ [eq. S7]

where Pc is the proportionality constant existing between the area of the ^{1}H NMR signals and the number of protons that generate them, A_K, A_L, A_{I+J} and A_F are the areas of the corresponding signals indicated in Table S2 and Figure 1, and $A_{4.26\text{-}4.38}$ and $A_{4.04\text{-}4.38}$ represent the areas of the signals between 4.26 and 4.38 ppm, and between 4.04 and 4.38 ppm, respectively.

Using these equations, the molar percentages of the different kinds of glycerides in relation to the total number of moles of glyceryl structures present (NT_{GS}) were determined as follows:

$NT_{GS}=N_{TG}+N_{1,2\text{-}DG}+N_{1,3\text{-}DG}+N_{2\text{-}MG}+N_{1\text{-}MG}+N_{Gol}$ [eq. S8]

$G\%=100N_G/NT_{GS}$ [eq. S9]

where G is each kind of glyceride (TG, 1,2-DG, 1,3-DG, 2-MG and 1-MG) and N_G the respective number of moles.

$Gol\%=100N_{Gol}/NT_{GS}$ [eq. S10]

B. Estimation of the molar percentage of linoleic FA plus AG (FA+AG). The molar percentage of linoleic (L%), AG plus FA, in relation to the total number of moles of AG plus FA (NT_{AG+FA}) present in the starting oils and in the lipid extracts of the corresponding digestates was estimated as follows:

$L\% = 100 * A_G / A_F$ [eq. S11]

where A_G and A_F are the areas of signals G and F indicated in Table S3 and Figure 1.

C. Estimation of the concentration of specific compounds in oil samples and in the lipids extract from digestates

The concentration of the several kinds of specific compounds (SC), expressed as millimoles per mole of the sum of AG+FA, was estimated by using the following equations:

$[SC] = [(A_{SC}/n)/(A_F/2)]*1000$ [eq. S12]

where A_{SC}, is the area of the signal selected for the quantification of each specific compound present in the sample, n the number of protons that generate the signals given in Tables S4 , S5 and Figure 2 and A_F is the area of the signal F in Table S3 and in Figure 1.

REFERENCES

Baker, J.K.; Myers, C.W. One-dimensional and two-dimensional ^{1}H-and ^{13}C-nuclear magnetic resonance (NMR) analysis of vitamin E raw materials or analytical reference standards. *Pharm Res* **1991**, *8*(6), 763-770.

Dufour, C.; Loonis, M. Regio-and stereoselective oxidation of linoleic acid bound to serum albumin: identification by ESI–mass spectrometry and NMR of the oxidation products. *Chem Phys Lipids* **2005**, *138*(1), 60-68.

Guillén, M.D.; Goicoechea, E. Detection of primary and secondary oxidation products by Fourier transform infrared spectroscopy (FTIR) and ^{1}H nuclear magnetic resonance (NMR) in sunflower oil during storage. *J Agr Food Chem* **2007**, 55(26), 10729-10736.

Guillén, M.D.; Ruiz, A. Oxidation process of oils with high content of linoleic acyl groups and formation of toxic hydroperoxy- and hydroxyalkenals. A study by ^{1}H nuclear magnetic resonance. *J Sci Food Agric* **2005**, 85(14), 2413-2420.

Guillén, M.D.; Ruiz, A. Rapid simultaneous determination by proton NMR of unsaturation and composition of acyl groups in vegetable oils. *Eur J Lipid Sci Technol* **2003**, *105*(11), 688-696.

Guillén, M.D.; Uriarte, P.S. Study by ^{1}H NMR spectroscopy of the evolution of extra virgin olive oil composition submitted to frying temperature in an industrial fryer for a prolonged period of time. *Food Chem* **2012**, *134*(1), 162-172.

Lin, D.; Zhang, J.; Sayre, L.M. Synthesis of six epoxyketooctadecenoic acid (EKODE) isomers, their generation from nonenzymatic oxidation of linoleic acid, and their reactivity with imidazole nucleophiles. *J Org Chem*, **2007**, *72*(25), 9471-9480.

Nieva-Echevarría, B.; Goicoechea, E.; Manzanos, M.J.; Guillén, M.D. A method based on ^{1}H NMR spectral data useful to evaluate the hydrolysis level in complex lipid mixtures. *Food Res Int* **2014**, 66, 379-387.

Ramsden, C.E.; Domenichiello, A.F.; Yuan, Z.X.; Sapio, M.R.; Keyes, G.S.; Mishra, S.K.; ... Davis, J.M. A systems approach for discovering linoleic acid derivatives that potentially mediate pain and itch. *Sci Signal* **2017**, 10(493), eaal5241. doi: 10.1126/scisignal.aal5241

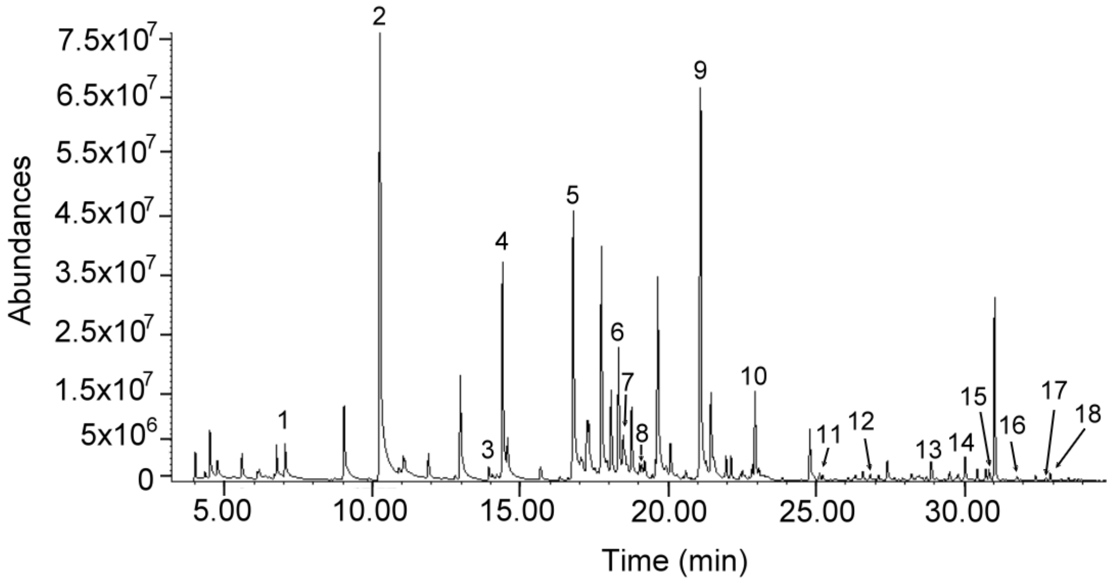

Figure S2. Region between 4-34 min of the total ion chromatogram obtained by SPME-GC/MS of the digestate of corn oil sample enriched in *alpha*-tocopherol DC5αT. Peaks identified: **(1)** pentanal; **(2)** hexanal; **(3)** furan, 2-butyl; **(4)** heptanal; **(5)** (*E*)-2-heptenal; **(6)** furan, 2-pentyl; **(7)** (*Z*,*E*)-2,4-heptadienal; **(8)** (*E*,*E*)-2,4-heptadienal; **(9)** (*E*)-2-octenal; **(10)** nonanal; **(11)** (*E*)-2-nonenal; **(12)** decanal; **(13)** (*E*)-2-decenal; **(14)** (*Z*,*E*)-2,4-decadienal; **(15)** (*E*,*E*)-2,4-decadienal; **(16)** 5-pentyl-2(5H)-furanone; **(17)** 4,5-epoxy-2-decenal (isomer); **(18)** 4,5-epoxy-(*E*)-2-decenal.
